# Frequency and Etiology of Pancytopenia in Patients Admitted to a Tertiary Care Hospital in Karachi

**DOI:** 10.7759/cureus.11057

**Published:** 2020-10-20

**Authors:** Rabia Farooque, Sundus Iftikhar, Fivzia Herekar, Muhammad Junaid Patel

**Affiliations:** 1 Internal Medicine, Liaquat University of Medical and Health Sciences, Jamshoro, PAK; 2 Internal Medicine, The Indus Hospital, Karachi, PAK; 3 Statistics, Indus Hospital Research Center, The Indus Hospital, Karachi, PAK; 4 Internal Medicine and Infectious Diseases, The Indus Hospital, Karachi, PAK

**Keywords:** pancytopenia

## Abstract

Introduction

Pancytopenia is an important hematologic problem encountered frequently in clinical practice characterized by a reduction in all three peripheral blood cell lineages, i.e., anemia, leucopenia, and thrombocytopenia, caused by myriad disease processes. Our study aimed to determine the frequency and etiology of pancytopenia in patients admitted under internal medicine services in a tertiary care hospital.

Method

This cross-sectional study was conducted in the in-patient internal medicine department, The Indus Hospital (TIH), Karachi, included 258 patients. To be eligible, participants had to give informed consent, be 14 years or older, and of either sex. The study involved a 20-30-minute interaction with the patient, involving an interview and physical examination, and access to electronic health record data.

Results

Out of 258 patients studied, 24 (9.3%) were diagnosed with pancytopenia, the male to female ratio was 1:1, no significant difference was observed in the proportion of ethnicity, religion, previous treatment, known infectious disease, and personal and occupational exposure among pancytopenic patients and other non-pancytopenic patients. Fever (n=14, 58.3%) was most common presenting complaint followed by fatigue (n=13, 54.2%) and weight loss (n=7, 29.2%) while most common signs were pallor (87.5% n=21), hepatomegaly (29.2%, n=7), and splenomegaly (25%, n=6). The most common cause of pancytopenia was megaloblastic anemia (n=10, 41.7%), followed by hypersplenism (n=4, 16.6%), acute infectious diseases (n=3, 12.5%), and autoimmune diseases (n=3, 12.5%).

Conclusion

Our study suggests that pancytopenia is a common finding among our patient population and a larger proportion has a treatable cause, thus carrying a favorable prognosis.

## Introduction

Pancytopenia is an important hematologic problem encountered frequently in clinical practice [1]. It’s defined as a reduction in all three peripheral blood cell lineages, i.e., anemia, leucopenia, and thrombocytopenia [2]. It’s not a disease entity itself but a presentation caused by diverse disease processes affecting bone marrow and/or peripheral cell lines [3-4]. Clinical presentation is related to the severity of cytopenias, leading to common presenting symptoms, including generalized weakness, shortness of breath, fever, weight loss, bleeding, etc. The management and prognosis of pancytopenia depend upon its severity and underlying etiology [5]. Its etiology ranges from benign conditions, such as nutritional deficiencies, infection, and drug effects, to malignant diseases such as lymphomas and leukemias [6]. Thus, identifying the correct etiology is of crucial importance in formulating therapeutic plans [7]. Its etiology is influenced by geography, socio-economic conditions, and endemic illnesses [8]. Nutritional megaloblastic anemia, caused by folate or vitamin B12 deficiency, is one of the leading causes of pancytopenia in developing countries, as it’s readily correctable and so should be suspected in patients with unexplained pancytopenia, macrocytosis, hypersegmented neutrophils, and neurological signs and symptoms [4,6]. There's a scarcity of data regarding the prevalence of pancytopenia in Pakistan. Therefore, we aimed to determine the frequency of pancytopenia and to evaluate the clinical features and underlying etiology of pancytopenia in the adult population admitted under internal medicine services in a tertiary care hospital.

## Materials and methods

This prospective cross-sectional study was conducted at The Indus Hospital, Karachi (TIH), a free-of-cost tertiary-care facility. A priori sample size of 258 was calculated using OpenEpi software (www.OpenEpi.com) with the following assumptions: prevalence of pancytopenia 21.2% [9], 5% desired precision, and 95% confidence interval. The institutional review board of The Indus Hospital approved all study protocols. Patients were recruited from the in-patient internal medicine department over a four-month period between July 2018 and October 2018. Patients admitted under internal medicine services at TIH, age 14 years and above, of either gender, and giving informed consent either themselves or their guardians if age was below 18 years were included in the study. Of 258 patients recruited from the internal medicine department, none was excluded, as no patient refused to participate in the study. Data on demographics, presenting symptoms, and medical history regarding known co-morbidities or chronic infections, exposure to potentially toxic agents or radiation, and medication use were recorded. Physical examination was performed to assess pallor, rash, oral lesions, jaundice, lymphadenopathy, hepatomegaly, or splenomegaly.

Pancytopenia was defined by the complete blood count report, as all peripheral blood lineages decreased below the normal reference range, based on criteria defined by De Gruchy [10] as follows:

Hemoglobin (Hb) level - <13.5 g/dL for males and <11.5 g/dL for females

Total leucocyte count (TLC) - <4 × 10ˆ9/L

Platelet (Plt) count - <150× 10ˆ9/L

Further workup to identify the etiology of pancytopenia was carried out as clinically indicated, which included reticulocyte count, serum lactate dehydrogenase (LDH), serum iron profile, vitamin B12 levels, red blood cell (RBC) folate, blood cultures, malarial parasite (MP), liver function test (LFT), chronic viral hepatitis serology, human immunodeficiency virus (HIV) screening serology, ultrasound and CT scan imaging, and bone marrow biopsy.

All the data were gathered on a predesigned questionnaire.

## Results

A total of 258 patients were enrolled in the study with a median age of 48 (28.8-65) years. Out of these, the majority (164; 63.6%) were females. One-hundred sixty-six (64.3%) of the patients were not working, followed by services and sales workers (n=88; 34.1%) and elementary occupation (n=34; 13.2%). Out of those who were not doing any kind of job, the majority were housewives (n=94; 56.6%), 22 (13.3%) were students, and 28 (16.9%) were dependent/bed-bound (Table 1).

**Table 1:** Demographic data

Variable	n(%)
Gender	
Female	164 (63.6)
Male	94 (36.4)
Age	
Median (IQR)	48 (28.8 - 65)
Min-Max	14 - 100
Occupation	
Not working	166 (64.3%)
Services and sales workers	88 (34.1)
Elementary occupation	34 (13.2)
Craft and related trades workers	14 (5.4
Plant and machinery operator	11 (4.3)
Professionals	9 (3.5)
Technicians and associate professionals	8 (3.1)
Skilled agricultural, forestry, and fishery workers	4 (1.6)
Clerical support workers	1 (0.4)
Forces	1 (0.4)
Reasons for not working	
Housewife	94 (56.6)
Dependent/bed-bound	28 (16.9)
Student	22 (13.3)
Retired/pensioner	16 (9.6)
Unemployed	6 (3.6)

More than half of the patients had one or more co-morbidity, with hypertension (n=95; 36.8%) and diabetes mellitus (n=67; 26%) being the most commonly observed among the patients (Table 2).

**Table 2:** Medical history

Known Co-morbid Diseases		
None	114 (44.2)	
Hypertension (HTN)	95 (36.8)	
Diabetes mellitus (DM)	67 (26)	
Ischemic heart disease (IHD)	35 (13.6)	
Cerebrovascular accident (CVA)	16 (6.2)	
Chronic kidney disease (CDK)	15 (5.8)	
Connective tissue disease	11 (4.3)	
Chronic obstructive pulmonary disease (COPD)	8 (3.1)	
Chronic liver disease	5 (1.9)	
End-stage renal disease (ESRD)	5 (1.9)	
Asthma	4 (1.6)	
Known Psychiatric illness	2 (0.8)	
Malignancy	2 (0.8)	
Cirrhosis	1 (0.4)	
Inflammatory bowel disease (IBD)	1 (0.4)	
Hemoglobinopathies (Thalassemia)	1 (0.4)	
Known infectious diseases		
None	226 (87.6)	
Tuberculosis	18 (7)	
Chronic viral	12 (4.7)	
Human immunodeficiency virus (HIV)/acquired immunodeficiency syndrome (AIDS)	2 (0.8)	
Description of prescribed medication		
None	148 (57.4)	
Angiotensin-converting enzyme inhibitors (ACE)/angiotensin receptor blocker (ARBs)	41 (15.9)	
Oral hypoglycemics	36 (14)	
Antiplatelet agents	30 (11.6)	
Beta-blockers	23 (8.9)	
Calcium channel blocker (CCB)	20 (7.8)	
Gastrointestinal (acid suppressants)	14 (5.4)	
Thiazide/loop diuretics	12 (4.7)	
Steroids	11 (4.3)	
Statin	10 (3.9)	
Vasodilators (nitrates/hydralazine)	9 (3.5)	
Disease-modifying anti-rheumatic drugs (DMARDs)	7 (2.7)	
Analgesics	7 (2.7)	
Bronchodilators (inhaled/oral)	4 (1.6)	
Anticoagulant agents	3 (1.2)	
Anti-thyroid	3 (1.2)	
Anti-epileptics	3 (1.2)	
Alpha-blockers	3 (1.2)	
Anti-depressants	2 (0.8)	
Sedatives/hypnotics	2 (0.8)	
Anxiolytics	1 (0.4)	
Anti-neoplastic drugs	1 (0.4)	
Anti-tuberculosis therapy	1 (0.4)	
Nonprescription medication		
None	199 (77.1)	
Allopathic medication	25 9.7)	
Herbal medication	25 (9.7)	
Homeopathic medication	21 (8.1)	
Personal & occupation exposure		
None	242 (93.8)	
Alcohol	4 (1.6)	
Glue vapors	4 (1.6)	
Organic solvents	3 (1.2)	
Benzene	1 (0.4)	
Radiation	1 (0.4)	

Furthermore, three-fifths of the patients had monocytopenia (n=154; 59.7%), 47 (18.2%) had bicytopenia, and 24 (9.3%) had pancytopenia. Out of 154 monocytopenic patients, 149 (96.8%) had anemia while five (3.2%) had thrombocytopenia and none of the patients had leucopenia (Table 3).

**Table 3:** Cytopenia categories

Cytopenia categories	
Pancytopenia	24 (9.3)
Bicytopenia	47 (18.2)
Monocytopenia	154 (59.7)
Normal	33 (12.8)

Among pancytopenic patients, fever was the most common presenting complaint (n=14, 58.3%) followed by fatigue (n=13, 54.2%) and weight loss (n=7, 29.2%). Pallor was seen in 87.5% (n=21) while hepatomegaly was found in 29.2% (n=7), splenomegaly in 25% (n=6), jaundice, rash and oral ulcers in 8.3% each (n=2 each), and lymphadenopathy in 4.2% (n=1) (Table 4).

**Table 4:** Clinical features of pancytopenia

Clinical feature	Frequency (%)
Pallor	21 (87.5%)
Fever	14 (58.3%)
Fatigue	13 (54.2%)
Weight loss	7 (29.2%)
Hepatomegaly	7 (29.2%)
Splenomegaly	6 (25%)
Jaundice	2 (8.3%)
Rash	2 (8.3%)
Oral ulcers	2 (8.3%)
Lymphadenopathy	1 (4.2%)

The most common cause of pancytopenia was found to be megaloblastic anemia (n=10, 41.7%) followed by hypersplenism (n=4, 16.6%), acute infectious cause (n=3, 12.5%), and autoimmune diseases (n=3, 12.5%). Chronic Hodgkin lymphoma was diagnosed in one patient (4.1%) and one patient was found to have chronic kidney disease (CKD). Two patients had pancytopenia with no obvious cause (Table 5).

**Table 5:** Etiology of pancytopenia

Causes of Pancytopenia	No. of cases (%)
Megaloblastic anemia	10 (41.7%)
Hypersplenism	4 (16.7%)
Acute infectious disease	3 (12.5%)
Autoimmune disease	3 (12.5%)
Chronic kidney disease	1 (4.2%)
Chronic Hodgkin lymphoma	1 (4.2%)
None	2 (8.3%)

In the hypersplenism group, one patient had chronic liver disease (CLD) secondary to hepatitis C infection, two had non-B/C CLD, and one had isolated splenomegaly with no cause identified. The three patients had pancytopenia associated with infectious diseases; their etiology was fulminant hepatic failure along with hospital-acquired septicemia, enteric fever, and complicated malaria, respectively. While, in the autoimmune disease group, one patient had autoimmune hemolytic anemia, the second had small vessel vasculitis, and the third had systemic lupus erythematosus.

Additionally, the distribution of pancytopenia was equal in both the genders (p=0.182; Table 6), whereas, the proportion of B-12 deficiency was higher in pancytopenia patients as compared to the non-pancytopenia patients (40% vs 6.8%, p=0.001). No statistically significant association was seen between pancytopenia and ethnicity, religion, previous treatment, infectious disease, and personal and occupational exposure (Table 6).

**Table 6:** Association of pancytopenia with patients’ characteristics

	Pancytopenia			P-value
	No	Yes	Total	
Gender				
Female	152 (65)	12 (50)	164 (63.6)	0.182^†^
Male	82 (35)	12 (50)	94 (36.4)	
Total	234 (100)	24 (100)	258 (100)	
Vitamin B12 deficiency				
No	137 (93.2)	9 (60)	146 (90.1)	0.001^ⱡ**^
Yes	10 (6.8)	6 (40)	16 (9.9)	
Total	147 (100)	15 (100)	162 (100)	
Physical examination				
Pallor	141 (95.9)	21 (91.3)	162 (95.3)	0.000^†***^
Rash	1 (0.7)	2 (8.7)	3 (1.8)	
Oral lesions	3 (2)	2 (8.7)	5 (2.9)	
Lymphadenopathy	4 (2.7)	1 (4.3)	5 (2.9)	
Hepatomegaly	22 (15)	7 (30.4)	29 (17.1)	
Jaundice	4 (2.7)	2 (8.7)	6 (3.5)	
Splenomegaly	6 (4.1)	6 (26.1)	12 (7.1)	
Previous treatment				
Transfusions	87 (91.6)	12 (100)	99 (92.5)	0.463^†^
Hematinic	19 (20)	1 (8.3)	20 (18.7)	
known infection disease				
Tuberculosis	16 (55.2)	2 (66.7)	18 (56.3)	0.947^†^
HIV/AIDS	2 (6.9)	0 (0)	2 (6.3)	
Chronic viral	11 (37.9)	1 (33.3)	12 (37.5)	
Personal and occupational exposure				
Alcohol	4 (28.6)	0 (0)	4 (25)	0.771^†^
Benzene	1 (7.1)	0 (0)	1 (6.3)	
Glue vapors	3 (21.4)	1 (50)	4 (25)	
Pesticide	0 (0)	0 (0)	0 (0)	
Organic solvents	2 (14.3)	1 (50)	3 (18.8)	
Radiation	1 (7.1)	0 (0)	1 (6.3)	
Presenting symptoms				
Fever	138 (61.1)	14 (58.3)	152 (60.8)	0.002^†**^
Night sweats	11 (4.9)	0 (0)	11 (4.4)	
Weight loss	38 (16.8)	7 (29.2)	45 (18)	
Fatigue	84 (37.2)	13 (54.2)	97 (38.8)	
Chest pain	16 (7.1)	1 (4.2)	17 (6.8)	
Bleeding	14 (6.2)	3 (12.5)	17 (6.8)	
Jaundice	3 (1.3)	4(16.7)	7 (2.8)	
Nausea/vomiting	66 (29.2)	5 (20.8)	71 (28.4)	
Shortness of breath	12(5.3%)	3 (12.5%)	15 (6)	
*P-value<0.05, **P-value<0.0001, †Pearson chi-square test, ⱡ Fisher's exact test				

Out of 24 pancytopenic patients, 18 patients were discharged with follow-up advised in medical outpatient services, three were referred to gastroenterology services, and one was referred to hematology services while two patients expired during the hospital stay.

## Discussion

Pancytopenia is a hematologic entity frequently encountered in clinical practice. In this study, the incidence of pancytopenia was 9.3% in patients admitted under internal medicine services while in other studies, its frequency is quite variable, and most of these studies are conducted on pediatric patients. A study conducted at Kuwait Teaching Hospital, Peshawar, showed its frequency to be 57.4% in the adult medicine department in 2015 [11] while another study conducted in the pathology department, Rawalpindi, Pakistan determined the frequency of pancytopenia in the combined adult and pediatric population to be 21.2% [9]. Umbreen et al. [7] and Shazia et al. [12] observed a 2.52% and 3.57% frequency of pancytopenia, respectively, in the pediatric population. Overall, these studies showed that pancytopenia is more common among the adult population in Pakistan.

We found an equal proportion of pancytopenia between both genders, that is, comparable to Osama et al. [13], whereas the Yaseen et al. study [3] showed that females constituted 62% of the pancytopenic patients while most other studies showed a male preponderance. Samreen et al. [14] observed a male-to-female ratio of 1.8:1 while Umbreen et al. [9] and Ikram et al [6] reported this to be 2.5:1 and 2:1, respectively.

Furthermore, we observed that pancytopenic patients were younger, with a median age of 38.8 years, which is consistent with Samreen et al. [14], Tariq et al. [15], and Hayat et al. [16].

In our study, we found megaloblastic anemia as the most common cause of pancytopenia, accounting for 41.7%. Osama et al. [13], Yaseen et al. [3], Aziz et al. [17], and studies from India had similar observations [5,18-21]. All patients in our study with megaloblastic anemia had a vitamin B 12 deficiency that is easily correctable, hence it should be suspected early on the basis of the megaloblastic picture on peripheral smear and mean corpuscular volume above 100 fL [13].

Hypersplenism accounted for 16.7% of cases of pancytopenia in this study while Hayat et al. [16], Osama et al. [13], and Ikram et al. [6] observed this to be 15.3%,19%, and 25%, respectively. In our study, three out of four patients had hypersplenism because of liver cirrhosis, one had isolated splenomegaly with no cause identified, and only one had cirrhosis secondary to chronic hepatitis C infection. This is in contrast to the very high burden of chronic liver disease due to viral hepatitis in Pakistan [22].

In our study, 12.5% of patients with pancytopenia had acute infectious etiology; while Osama et al. [13] and Umbreen et al. [9] reported similar observations, other studies had widely different observations regarding infectious etiology [1-2,5,14,23-25].

Autoimmune diseases caused pancytopenia in 12.5% of patients. Out of three, one patient had autoimmune hemolytic anemia, the second had small vessel vasculitis, and the third had systemic lupus erythematosus. Osama et al. [13], Khan et al [23], and Hanan et al. [26] reported 3%, 1.3%, and 11.2% cases of pancytopenia, respectively, caused by autoimmune diseases.

Chronic Hodgkin lymphoma was diagnosed in one patient (4.1%); other studies observed malignant conditions more frequently than this [2,9,13-14,16-17].

Chronic kidney disease (CKD) was found in one patient, and though it is more frequently associated with microcytic iron deficiency anemia, it can cause pancytopenia via hyperparathyroidism. CKD is associated with secondary and tertiary hyperparathyroidism, which can lead to myelofibrosis [26-27].

## Conclusions

In this study, we found that pancytopenia is a common finding among our adult population, and a larger proportion had a treatable cause, thus carrying a favorable prognosis. It also emphasizes the need for an accurate diagnosis that can facilitate timely treatment and impact morbidity and mortality.
